# Supplementary material for: Plasma Gelsolin Inhibits Natural Killer Cell Function and Confers Chemoresistance in Epithelial Ovarian Cancer
Source: Cells. 2024 May 24;13(11):905. doi: 10.3390/cells13110905 (PMC11171658; doi:10.3390/cells13110905)
Supplement: Supplementary file 1 [file cells-13-00905-s001.zip › Sup_Fig_GSN_NK_20240515.pptx]

## Slide 1
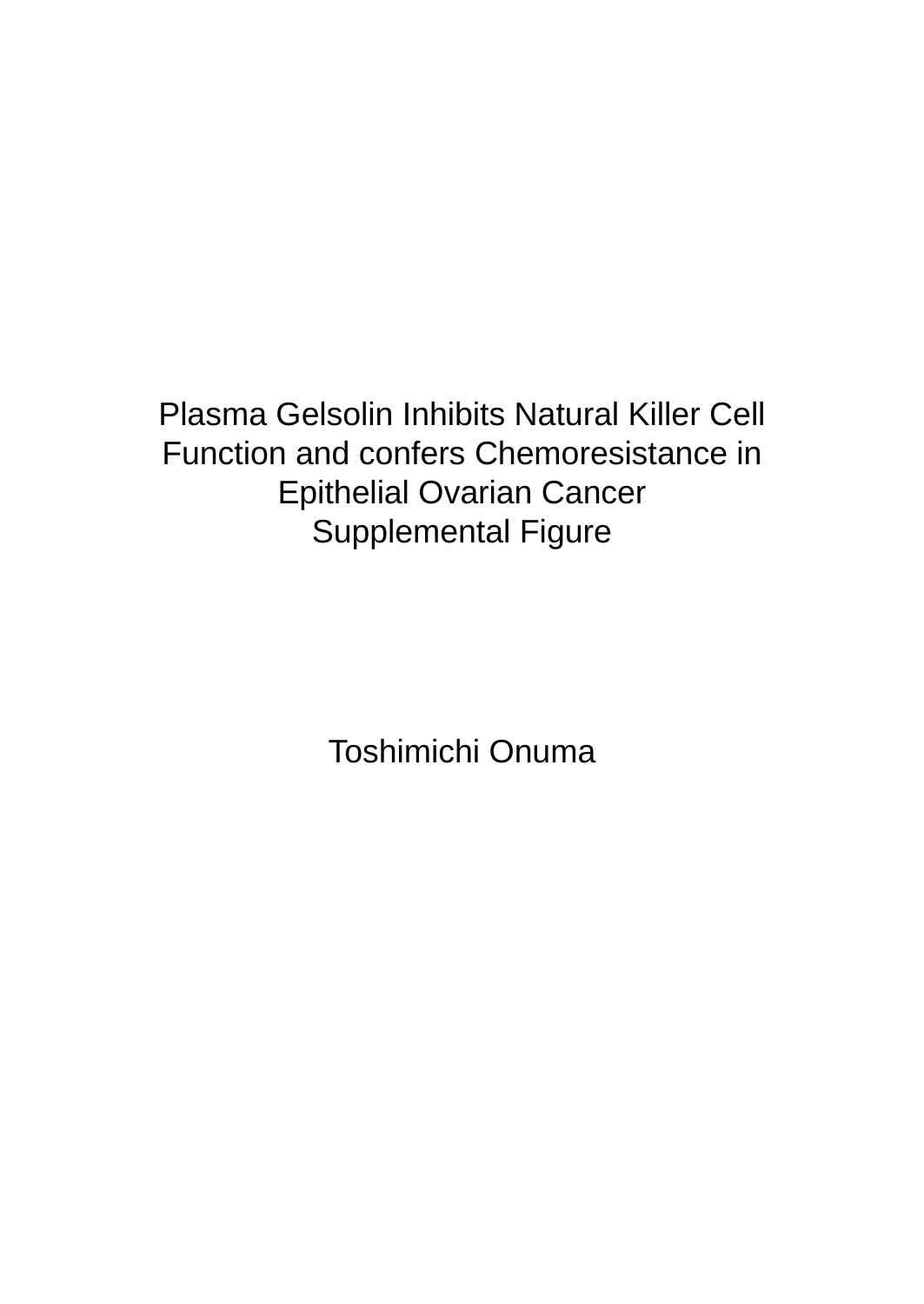

Plasma Gelsolin Inhibits Natural Killer Cell Function and confers Chemoresistance in Epithelial Ovarian Cancer
Supplemental Figure
Toshimichi Onuma

## Slide 2
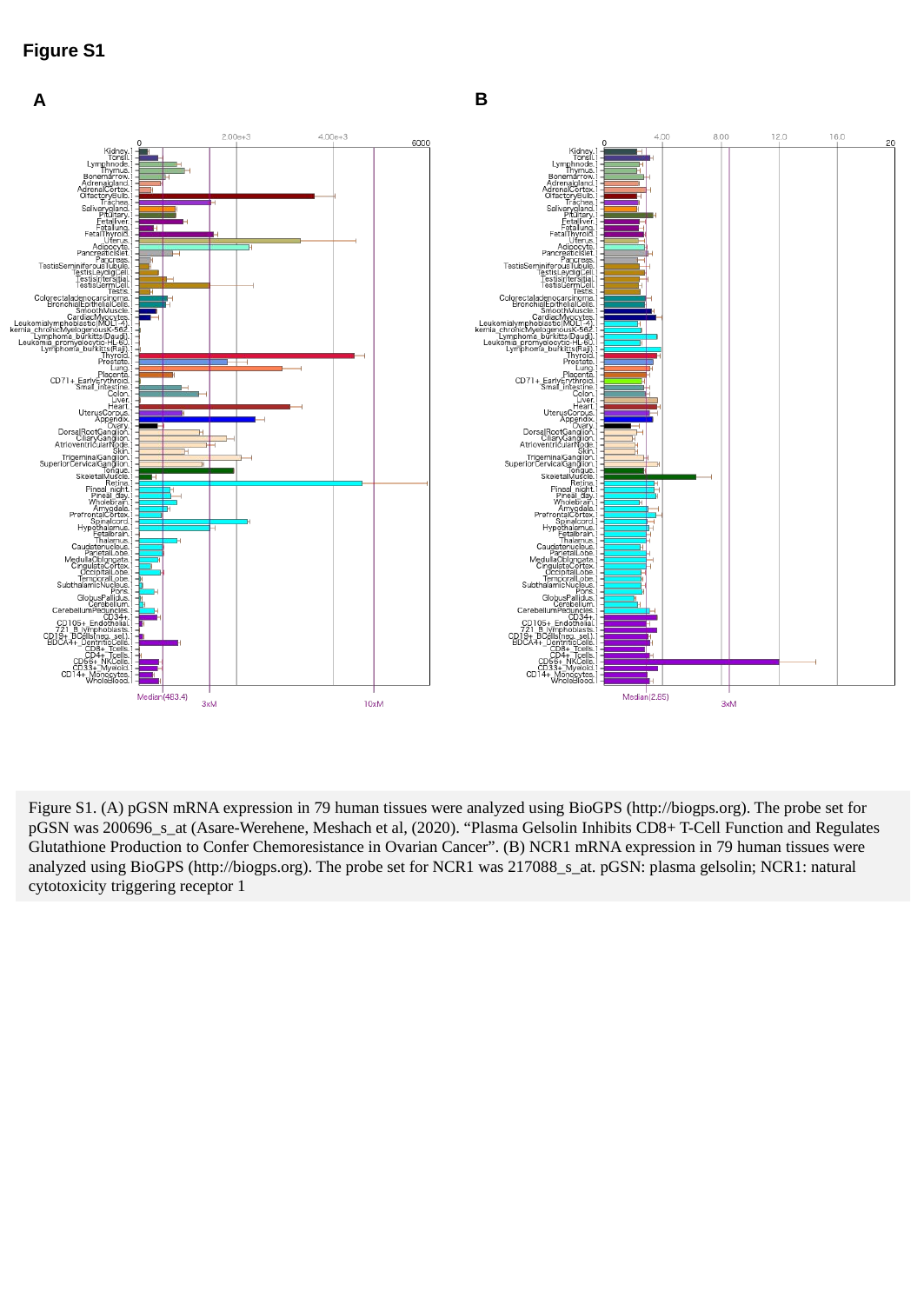

Figure S1
B
A
Figure S1. (A) pGSN mRNA expression in 79 human tissues were analyzed using BioGPS (http://biogps.org). The probe set for pGSN was 200696_s_at (Asare-Werehene, Meshach et al, (2020). “Plasma Gelsolin Inhibits CD8+ T-Cell Function and Regulates Glutathione Production to Confer Chemoresistance in Ovarian Cancer”. (B) NCR1 mRNA expression in 79 human tissues were analyzed using BioGPS (http://biogps.org). The probe set for NCR1 was 217088_s_at. pGSN: plasma gelsolin; NCR1: natural cytotoxicity triggering receptor 1

## Slide 3
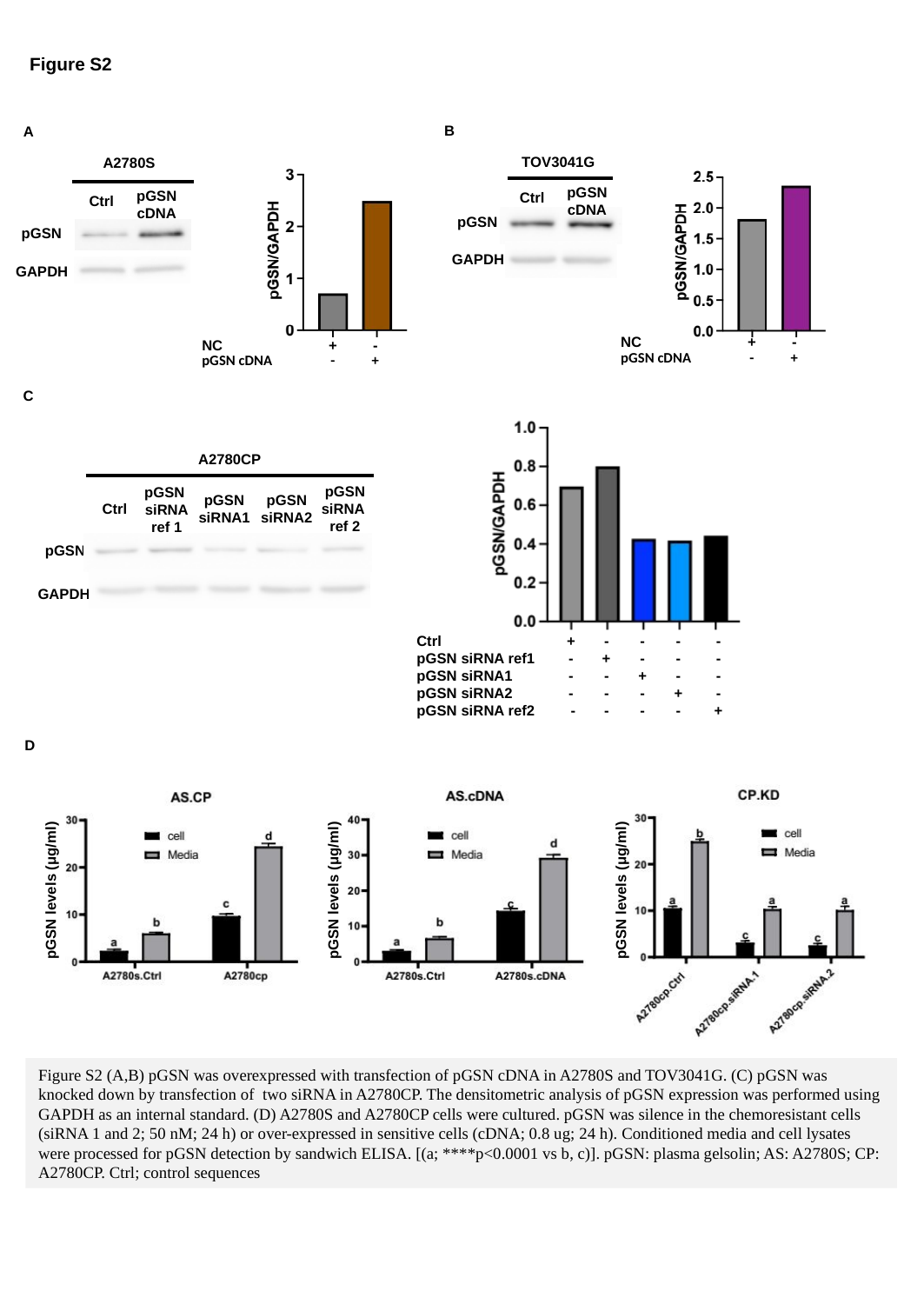

Figure S2
B
A
TOV3041G
A2780S
pGSN cDNA
pGSN cDNA
Ctrl
Ctrl
pGSN
pGSN
GAPDH
GAPDH
| NC | + | - |
| --- | --- | --- |
| pGSN cDNA | - | + |
| NC | + | - |
| --- | --- | --- |
| pGSN cDNA | - | + |
C
A2780CP
pGSN siRNA
ref 2
pGSN
siRNA
ref 1
pGSN siRNA1
pGSN siRNA2
Ctrl
pGSN
GAPDH
| Ctrl | + | - | - | - | - |
| --- | --- | --- | --- | --- | --- |
| pGSN siRNA ref1 | - | + | - | - | - |
| pGSN siRNA1 | - | - | + | - | - |
| pGSN siRNA2 | - | - | - | + | - |
| pGSN siRNA ref2 | - | - | - | - | + |
D
pGSN levels (μg/ml)
pGSN levels (μg/ml)
pGSN levels (μg/ml)
Figure S2 (A,B) pGSN was overexpressed with transfection of pGSN cDNA in A2780S and TOV3041G. (C) pGSN was knocked down by transfection of two siRNA in A2780CP. The densitometric analysis of pGSN expression was performed using GAPDH as an internal standard. (D) A2780S and A2780CP cells were cultured. pGSN was silence in the chemoresistant cells (siRNA 1 and 2; 50 nM; 24 h) or over-expressed in sensitive cells (cDNA; 0.8 ug; 24 h). Conditioned media and cell lysates were processed for pGSN detection by sandwich ELISA. [(a; ****p<0.0001 vs b, c)]. pGSN: plasma gelsolin; AS: A2780S; CP: A2780CP. Ctrl; control sequences

## Slide 4
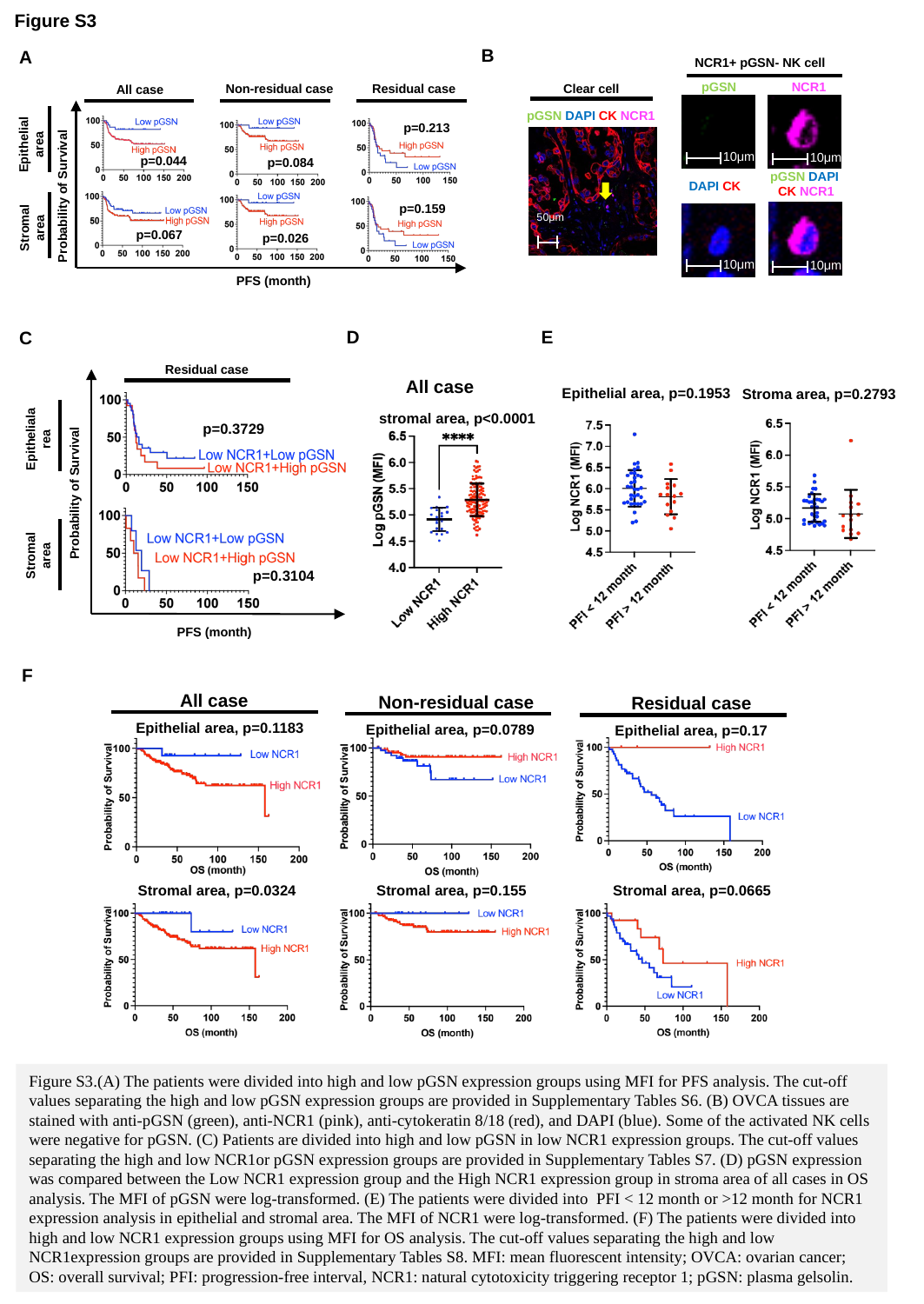

Figure S3
B
A
NCR1+ pGSN- NK cell
pGSN
NCR1
Clear cell
pGSN DAPI CK NCR1
10μm
10μm
pGSN DAPI
CK NCR1
DAPI CK
50μm
10μm
10μm
Residual case
Non-residual case
All case
p=0.213
Epithelial area
p=0.044
p=0.084
Probability of Survival
p=0.159
Stromal area
p=0.067
p=0.026
PFS (month)
D
E
C
Residual case
All case
Epithelial area, p=0.1953
Stroma area, p=0.2793
stromal area, p<0.0001
p=0.3729
Epithelialarea
Probability of Survival
Stromal area
p=0.3104
PFS (month)
F
All case
Epithelial area, p=0.1183
Stromal area, p=0.0324
Non-residual case
Epithelial area, p=0.0789
Stromal area, p=0.155
Residual case
Epithelial area, p=0.17
Stromal area, p=0.0665
Figure S3.(A) The patients were divided into high and low pGSN expression groups using MFI for PFS analysis. The cut-off values separating the high and low pGSN expression groups are provided in Supplementary Tables S6. (B) OVCA tissues are stained with anti-pGSN (green), anti-NCR1 (pink), anti-cytokeratin 8/18 (red), and DAPI (blue). Some of the activated NK cells were negative for pGSN. (C) Patients are divided into high and low pGSN in low NCR1 expression groups. The cut-off values separating the high and low NCR1or pGSN expression groups are provided in Supplementary Tables S7. (D) pGSN expression was compared between the Low NCR1 expression group and the High NCR1 expression group in stroma area of all cases in OS analysis. The MFI of pGSN were log-transformed. (E) The patients were divided into PFI < 12 month or >12 month for NCR1 expression analysis in epithelial and stromal area. The MFI of NCR1 were log-transformed. (F) The patients were divided into high and low NCR1 expression groups using MFI for OS analysis. The cut-off values separating the high and low NCR1expression groups are provided in Supplementary Tables S8. MFI: mean fluorescent intensity; OVCA: ovarian cancer; OS: overall survival; PFI: progression-free interval, NCR1: natural cytotoxicity triggering receptor 1; pGSN: plasma gelsolin.

## Slide 5
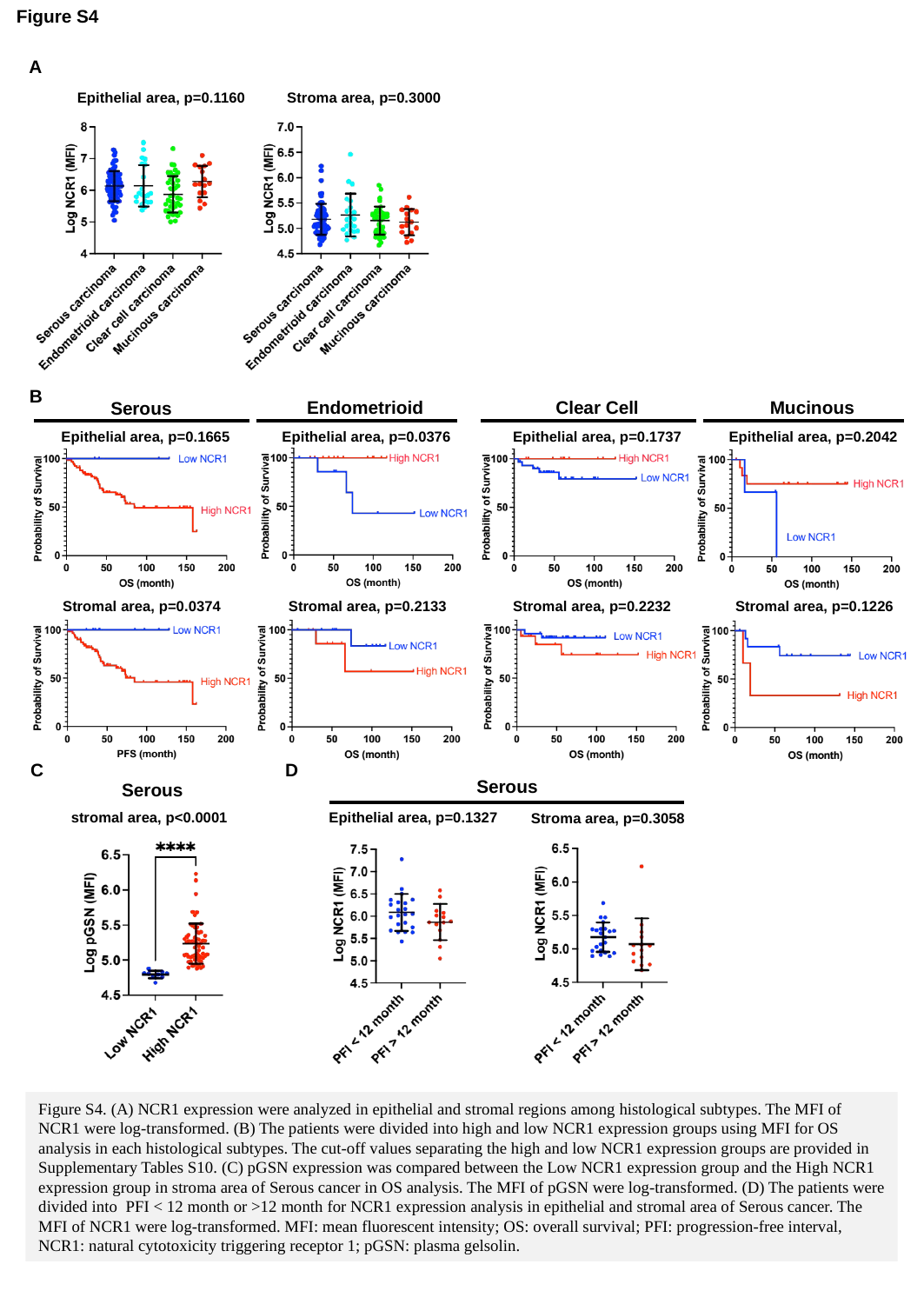

Figure S4
A
Epithelial area, p=0.1160
Stroma area, p=0.3000
B
Endometrioid
Epithelial area, p=0.0376
Stromal area, p=0.2133
Clear Cell
Epithelial area, p=0.1737
Stromal area, p=0.2232
Mucinous
Epithelial area, p=0.2042
Stromal area, p=0.1226
Serous
Epithelial area, p=0.1665
Stromal area, p=0.0374
D
C
Serous
Epithelial area, p=0.1327
Stroma area, p=0.3058
Serous
stromal area, p<0.0001
Figure S4. (A) NCR1 expression were analyzed in epithelial and stromal regions among histological subtypes. The MFI of NCR1 were log-transformed. (B) The patients were divided into high and low NCR1 expression groups using MFI for OS analysis in each histological subtypes. The cut-off values separating the high and low NCR1 expression groups are provided in Supplementary Tables S10. (C) pGSN expression was compared between the Low NCR1 expression group and the High NCR1 expression group in stroma area of Serous cancer in OS analysis. The MFI of pGSN were log-transformed. (D) The patients were divided into PFI < 12 month or >12 month for NCR1 expression analysis in epithelial and stromal area of Serous cancer. The MFI of NCR1 were log-transformed. MFI: mean fluorescent intensity; OS: overall survival; PFI: progression-free interval, NCR1: natural cytotoxicity triggering receptor 1; pGSN: plasma gelsolin.

## Slide 6
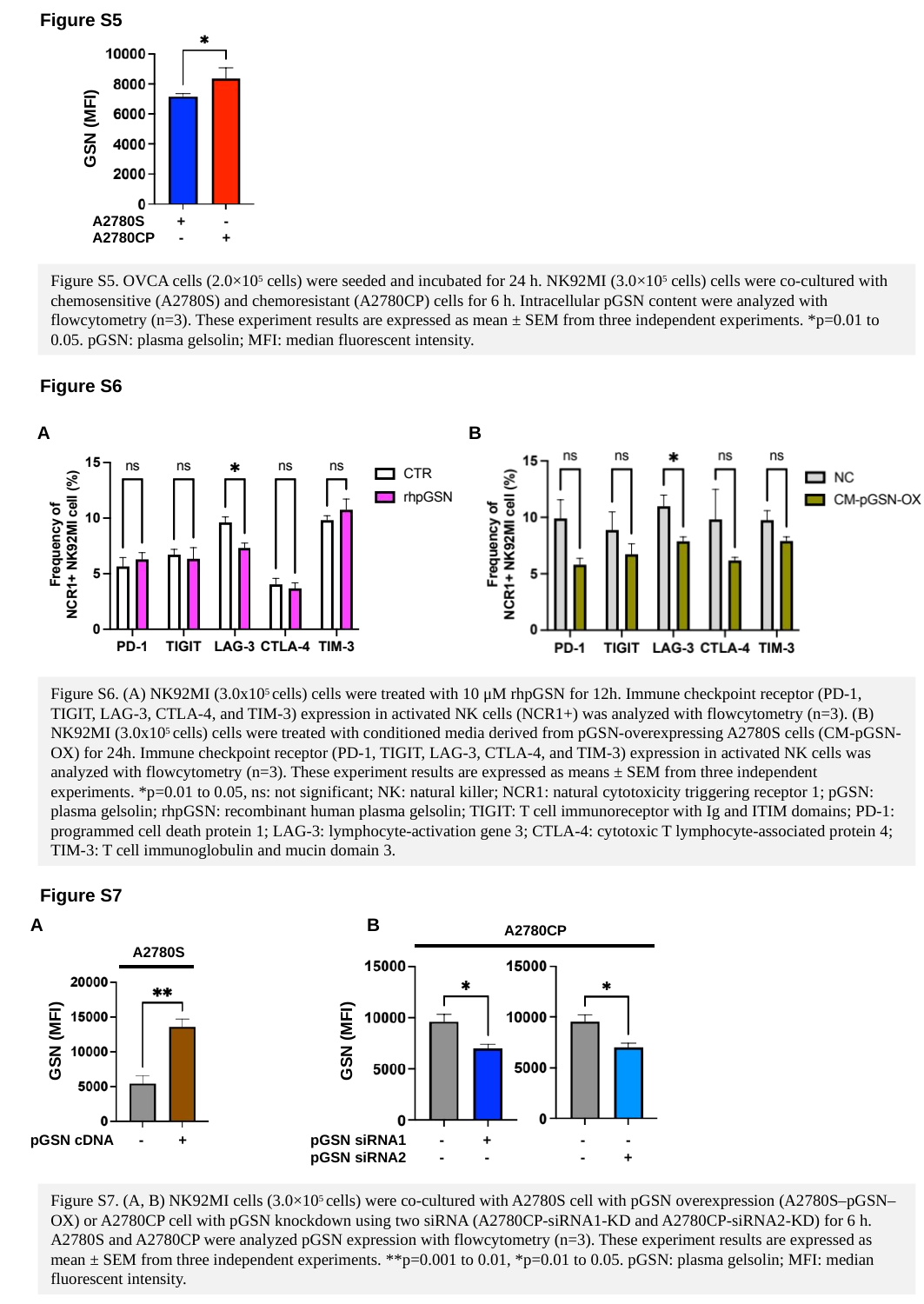

Figure S5
GSN (MFI)
| A2780S | + | - |
| --- | --- | --- |
| A2780CP | - | + |
Figure S5. OVCA cells (2.0×10^5^ cells) were seeded and incubated for 24 h. NK92MI (3.0×10^5^ cells) cells were co-cultured with chemosensitive (A2780S) and chemoresistant (A2780CP) cells for 6 h. Intracellular pGSN content were analyzed with flowcytometry (n=3). These experiment results are expressed as mean ± SEM from three independent experiments. *p=0.01 to 0.05. pGSN: plasma gelsolin; MFI: median fluorescent intensity.
Figure S6
A
B
Figure S6. (A) NK92MI (3.0x10^5^ cells) cells were treated with 10 μM rhpGSN for 12h. Immune checkpoint receptor (PD-1, TIGIT, LAG-3, CTLA-4, and TIM-3) expression in activated NK cells (NCR1+) was analyzed with flowcytometry (n=3). (B) NK92MI (3.0x10^5^ cells) cells were treated with conditioned media derived from pGSN-overexpressing A2780S cells (CM-pGSN-OX) for 24h. Immune checkpoint receptor (PD-1, TIGIT, LAG-3, CTLA-4, and TIM-3) expression in activated NK cells was analyzed with flowcytometry (n=3). These experiment results are expressed as means ± SEM from three independent experiments. *p=0.01 to 0.05, ns: not significant; NK: natural killer; NCR1: natural cytotoxicity triggering receptor 1; pGSN: plasma gelsolin; rhpGSN: recombinant human plasma gelsolin; TIGIT: T cell immunoreceptor with Ig and ITIM domains; PD-1: programmed cell death protein 1; LAG-3: lymphocyte-activation gene 3; CTLA-4: cytotoxic T lymphocyte-associated protein 4; TIM-3: T cell immunoglobulin and mucin domain 3.
Figure S7
A
B
 A2780CP
A2780S
GSN (MFI)
GSN (MFI)
| pGSN siRNA1 | - | + | | - | - |
| --- | --- | --- | --- | --- | --- |
| pGSN siRNA2 | - | - | | - | + |
| pGSN cDNA | - | + |
| --- | --- | --- |
Figure S7. (A, B) NK92MI cells (3.0×10^5^ cells) were co-cultured with A2780S cell with pGSN overexpression (A2780S–pGSN–OX) or A2780CP cell with pGSN knockdown using two siRNA (A2780CP-siRNA1-KD and A2780CP-siRNA2-KD) for 6 h. A2780S and A2780CP were analyzed pGSN expression with flowcytometry (n=3). These experiment results are expressed as mean ± SEM from three independent experiments. **p=0.001 to 0.01, *p=0.01 to 0.05. pGSN: plasma gelsolin; MFI: median fluorescent intensity.

## Slide 7
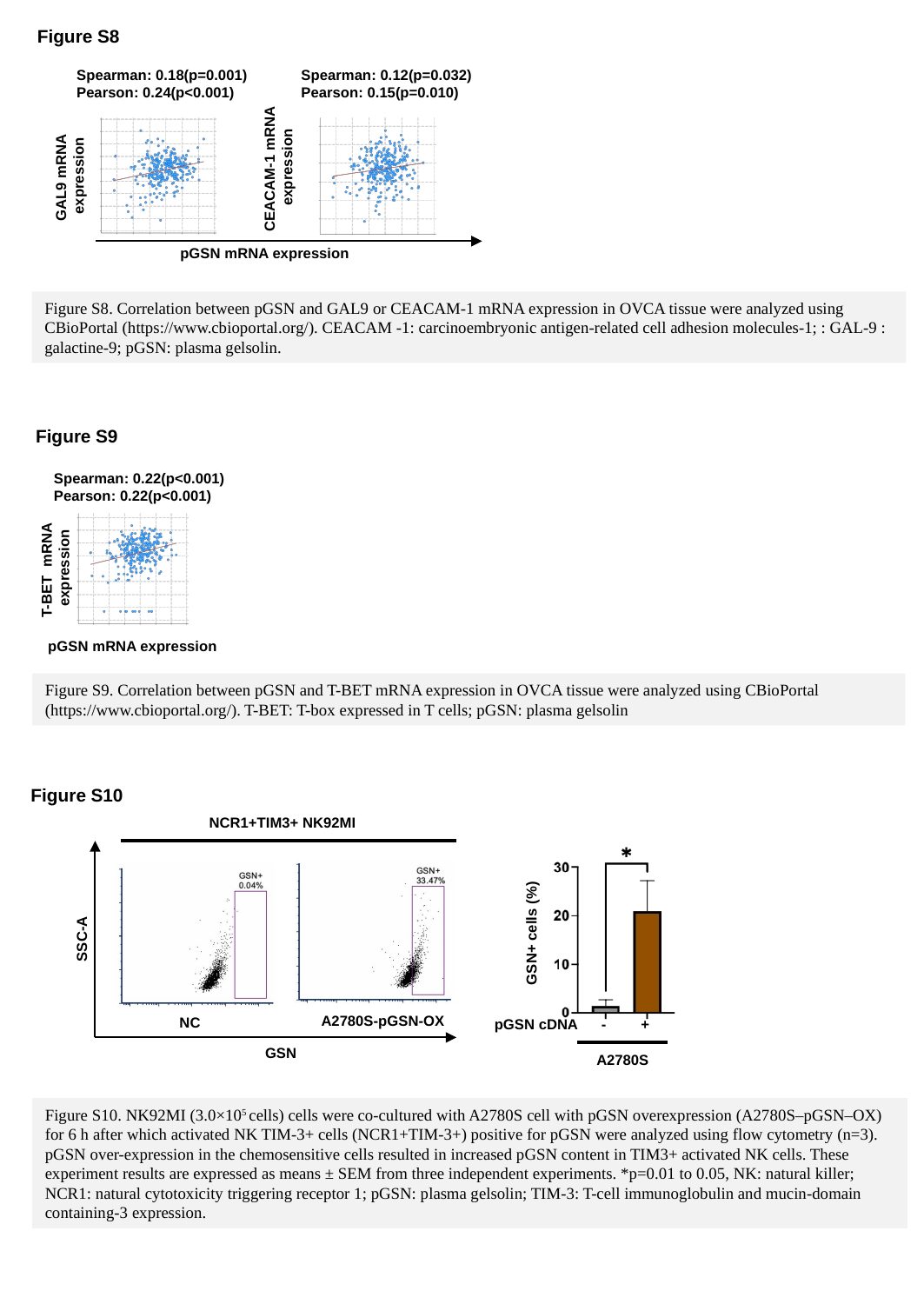

Figure S8
Spearman: 0.18(p=0.001)
Pearson: 0.24(p<0.001)
Spearman: 0.12(p=0.032)
Pearson: 0.15(p=0.010)
CEACAM-1 mRNA
 expression
GAL9 mRNA
expression
pGSN mRNA expression
Figure S8. Correlation between pGSN and GAL9 or CEACAM-1 mRNA expression in OVCA tissue were analyzed using CBioPortal (https://www.cbioportal.org/). CEACAM -1: carcinoembryonic antigen-related cell adhesion molecules-1; : GAL-9 : galactine-9; pGSN: plasma gelsolin.
Figure S9
Spearman: 0.22(p<0.001)
Pearson: 0.22(p<0.001)
T-BET mRNA
 expression
pGSN mRNA expression
Figure S9. Correlation between pGSN and T-BET mRNA expression in OVCA tissue were analyzed using CBioPortal (https://www.cbioportal.org/). T-BET: T-box expressed in T cells; pGSN: plasma gelsolin
Figure S10
 NCR1+TIM3+ NK92MI
SSC-A
 A2780S-pGSN-OX
 NC
 GSN
GSN+ cells (%)
| pGSN cDNA | - | + |
| --- | --- | --- |
A2780S
Figure S10. NK92MI (3.0×10^5^ cells) cells were co-cultured with A2780S cell with pGSN overexpression (A2780S–pGSN–OX) for 6 h after which activated NK TIM-3+ cells (NCR1+TIM-3+) positive for pGSN were analyzed using flow cytometry (n=3). pGSN over-expression in the chemosensitive cells resulted in increased pGSN content in TIM3+ activated NK cells. These experiment results are expressed as means ± SEM from three independent experiments. *p=0.01 to 0.05, NK: natural killer; NCR1: natural cytotoxicity triggering receptor 1; pGSN: plasma gelsolin; TIM-3: T-cell immunoglobulin and mucin-domain containing-3 expression.
