# Supplementary material for: Plasma Gelsolin Inhibits Natural Killer Cell Function and Confers Chemoresistance in Epithelial Ovarian Cancer
Source: Cells. 2024 May 24;13(11):905. doi: 10.3390/cells13110905 (PMC11171658; doi:10.3390/cells13110905)
Supplement: Supplementary file 1 [file cells-13-00905-s001.zip › Sup_table_pGSN_NK_20240516.pptx]

## Slide 1
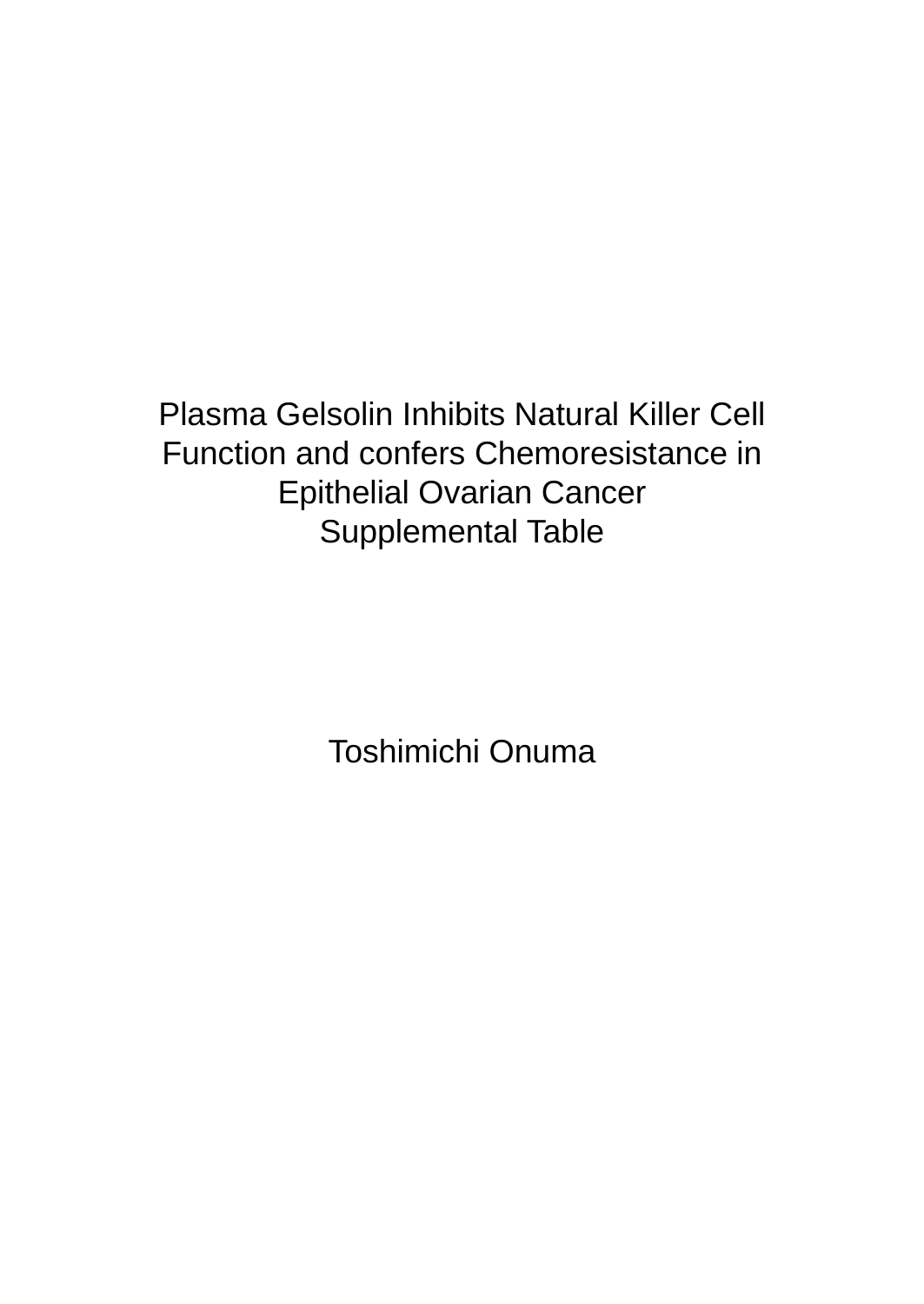

Plasma Gelsolin Inhibits Natural Killer Cell Function and confers Chemoresistance in Epithelial Ovarian Cancer
Supplemental Table
Toshimichi Onuma

## Slide 2
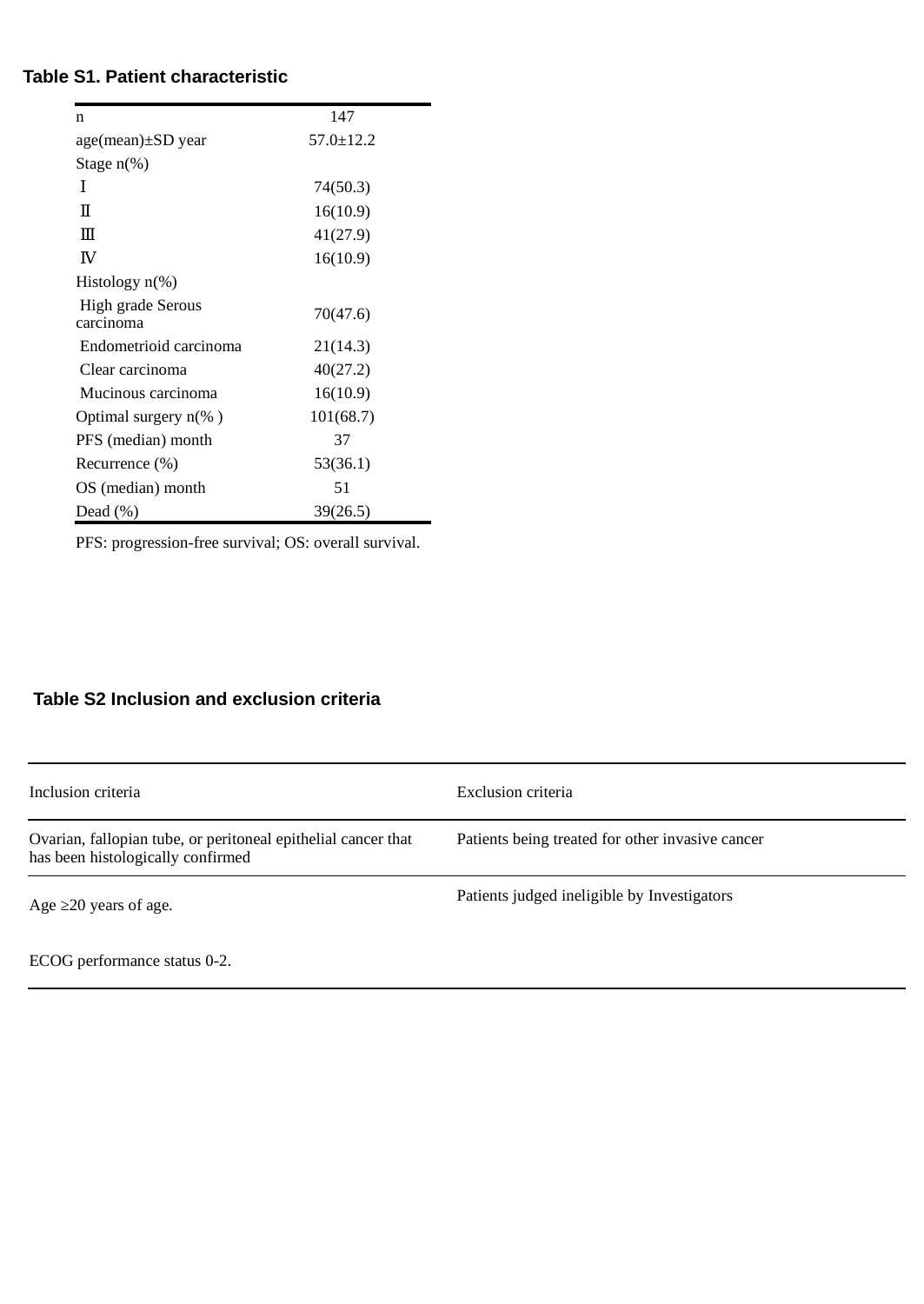

Table S1. Patient characteristic
| n | 147 |
| --- | --- |
| age(mean)±SD year | 57.0±12.2 |
| Stage n(%) | |
| Ⅰ | 74(50.3) |
| Ⅱ | 16(10.9) |
| Ⅲ | 41(27.9) |
| Ⅳ | 16(10.9) |
| Histology n(%) | |
| High grade Serous carcinoma | 70(47.6) |
| Endometrioid carcinoma | 21(14.3) |
| Clear carcinoma | 40(27.2) |
| Mucinous carcinoma | 16(10.9) |
| Optimal surgery n(% ) | 101(68.7) |
| PFS (median) month | 37 |
| Recurrence (%) | 53(36.1) |
| OS (median) month | 51 |
| Dead (%) | 39(26.5) |
PFS: progression-free survival; OS: overall survival.
Table S2 Inclusion and exclusion criteria
| Inclusion criteria | Exclusion criteria |
| --- | --- |
| Ovarian, fallopian tube, or peritoneal epithelial cancer that has been histologically confirmed | Patients being treated for other invasive cancer |
| Age ≥20 years of age. | Patients judged ineligible by Investigators |
| ECOG performance status 0-2. | |

## Slide 3
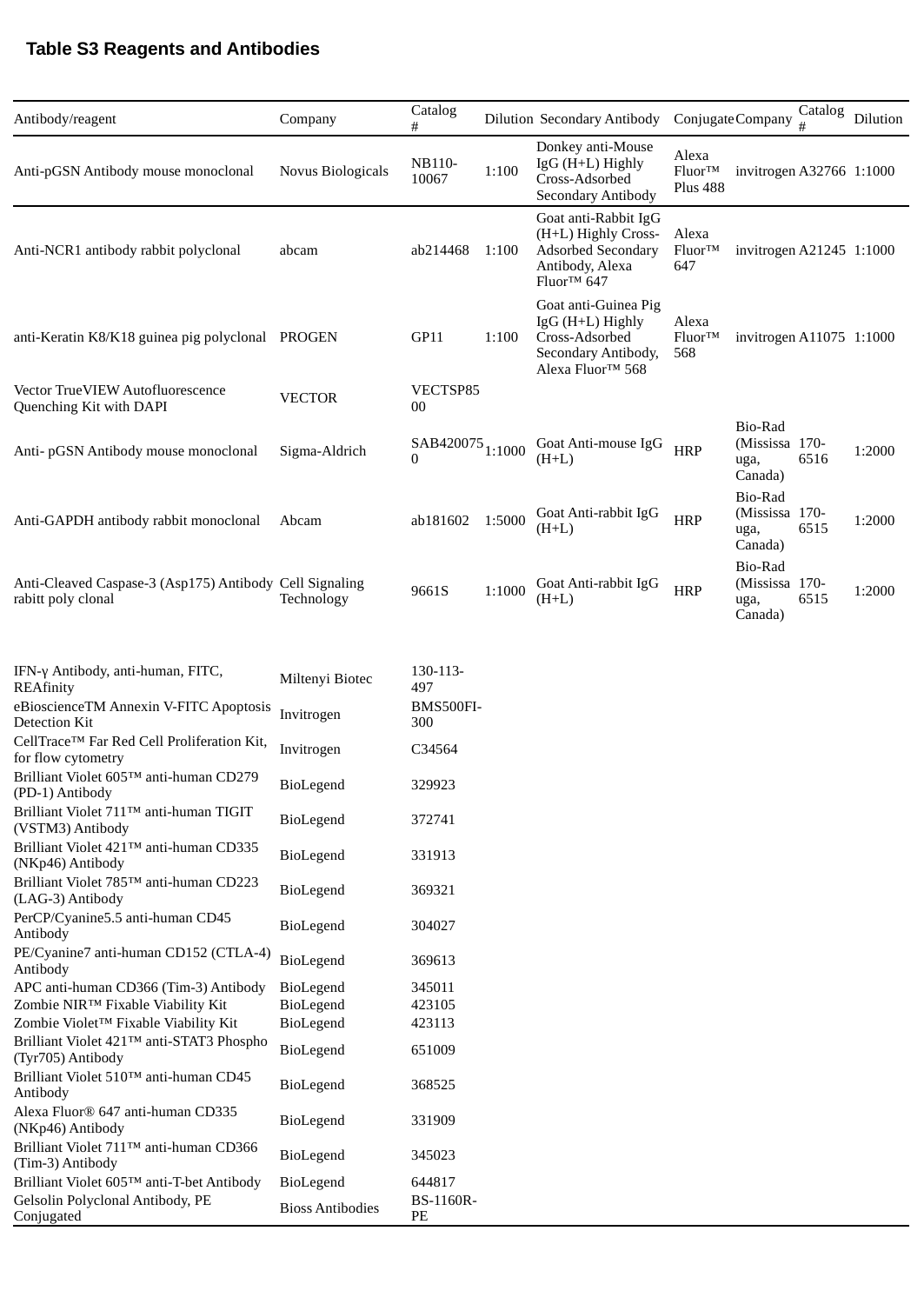

Table S3 Reagents and Antibodies
| Antibody/reagent | Company | Catalog # | Dilution | Secondary Antibody | Conjugate | Company | Catalog # | Dilution |
| --- | --- | --- | --- | --- | --- | --- | --- | --- |
| Anti-pGSN Antibody mouse monoclonal | Novus Biologicals | NB110-10067 | 1:100 | Donkey anti-Mouse IgG (H+L) Highly Cross-Adsorbed Secondary Antibody | Alexa Fluor™ Plus 488 | invitrogen | A32766 | 1:1000 |
| Anti-NCR1 antibody rabbit polyclonal | abcam | ab214468 | 1:100 | Goat anti-Rabbit IgG (H+L) Highly Cross-Adsorbed Secondary Antibody, Alexa Fluor™ 647 | Alexa Fluor™ 647 | invitrogen | A21245 | 1:1000 |
| anti-Keratin K8/K18 guinea pig polyclonal | PROGEN | GP11 | 1:100 | Goat anti-Guinea Pig IgG (H+L) Highly Cross-Adsorbed Secondary Antibody, Alexa Fluor™ 568 | Alexa Fluor™ 568 | invitrogen | A11075 | 1:1000 |
| Vector TrueVIEW Autofluorescence Quenching Kit with DAPI | VECTOR | VECTSP8500 | | | | | | |
| Anti- pGSN Antibody mouse monoclonal | Sigma-Aldrich | SAB4200750 | 1:1000 | Goat Anti-mouse IgG (H+L) | HRP | Bio-Rad (Mississauga, Canada) | 170-6516 | 1:2000 |
| Anti-GAPDH antibody rabbit monoclonal | Abcam | ab181602 | 1:5000 | Goat Anti-rabbit IgG (H+L) | HRP | Bio-Rad (Mississauga, Canada) | 170-6515 | 1:2000 |
| Anti-Cleaved Caspase-3 (Asp175) Antibody rabitt poly clonal | Cell Signaling Technology | 9661S | 1:1000 | Goat Anti-rabbit IgG (H+L) | HRP | Bio-Rad (Mississauga, Canada) | 170-6515 | 1:2000 |
| | | | | | | | | |
| | | | | | | | | |
| IFN-γ Antibody, anti-human, FITC, REAfinity | Miltenyi Biotec | 130-113-497 | | | | | | |
| eBioscienceTM Annexin V-FITC Apoptosis Detection Kit | Invitrogen | BMS500FI-300 | | | | | | |
| CellTrace™ Far Red Cell Proliferation Kit, for flow cytometry | Invitrogen | C34564 | | | | | | |
| Brilliant Violet 605™ anti-human CD279 (PD-1) Antibody | BioLegend | 329923 | | | | | | |
| Brilliant Violet 711™ anti-human TIGIT (VSTM3) Antibody | BioLegend | 372741 | | | | | | |
| Brilliant Violet 421™ anti-human CD335 (NKp46) Antibody | BioLegend | 331913 | | | | | | |
| Brilliant Violet 785™ anti-human CD223 (LAG-3) Antibody | BioLegend | 369321 | | | | | | |
| PerCP/Cyanine5.5 anti-human CD45 Antibody | BioLegend | 304027 | | | | | | |
| PE/Cyanine7 anti-human CD152 (CTLA-4) Antibody | BioLegend | 369613 | | | | | | |
| APC anti-human CD366 (Tim-3) Antibody | BioLegend | 345011 | | | | | | |
| Zombie NIR™ Fixable Viability Kit | BioLegend | 423105 | | | | | | |
| Zombie Violet™ Fixable Viability Kit | BioLegend | 423113 | | | | | | |
| Brilliant Violet 421™ anti-STAT3 Phospho (Tyr705) Antibody | BioLegend | 651009 | | | | | | |
| Brilliant Violet 510™ anti-human CD45 Antibody | BioLegend | 368525 | | | | | | |
| Alexa Fluor® 647 anti-human CD335 (NKp46) Antibody | BioLegend | 331909 | | | | | | |
| Brilliant Violet 711™ anti-human CD366 (Tim-3) Antibody | BioLegend | 345023 | | | | | | |
| Brilliant Violet 605™ anti-T-bet Antibody | BioLegend | 644817 | | | | | | |
| Gelsolin Polyclonal Antibody, PE Conjugated | Bioss Antibodies | BS-1160R-PE | | | | | | |

## Slide 4
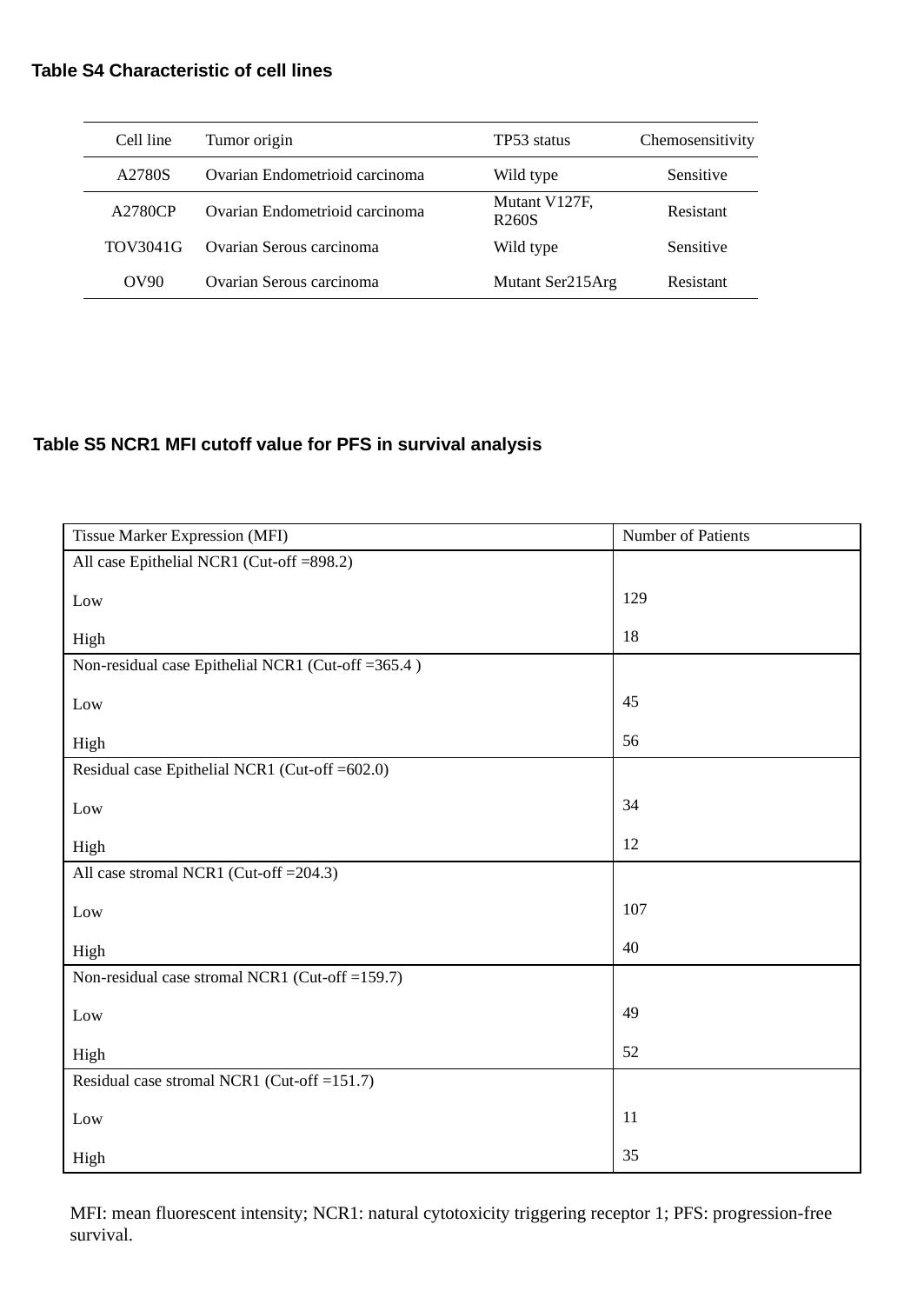

Table S4 Characteristic of cell lines
| Cell line | Tumor origin | TP53 status | Chemosensitivity |
| --- | --- | --- | --- |
| A2780S | Ovarian Endometrioid carcinoma | Wild type | Sensitive |
| A2780CP | Ovarian Endometrioid carcinoma | Mutant V127F, R260S | Resistant |
| TOV3041G | Ovarian Serous carcinoma | Wild type | Sensitive |
| OV90 | Ovarian Serous carcinoma | Mutant Ser215Arg | Resistant |
Table S5 NCR1 MFI cutoff value for PFS in survival analysis
| Tissue Marker Expression (MFI) | Number of Patients |
| --- | --- |
| All case Epithelial NCR1 (Cut-off =898.2) Low High | 129 18 |
| Non-residual case Epithelial NCR1 (Cut-off =365.4 ) Low High | 45 56 |
| Residual case Epithelial NCR1 (Cut-off =602.0) Low High | 34 12 |
| All case stromal NCR1 (Cut-off =204.3) Low High | 107 40 |
| Non-residual case stromal NCR1 (Cut-off =159.7) Low High | 49 52 |
| Residual case stromal NCR1 (Cut-off =151.7) Low High | 11 35 |
MFI: mean fluorescent intensity; NCR1: natural cytotoxicity triggering receptor 1; PFS: progression-free survival.

## Slide 5
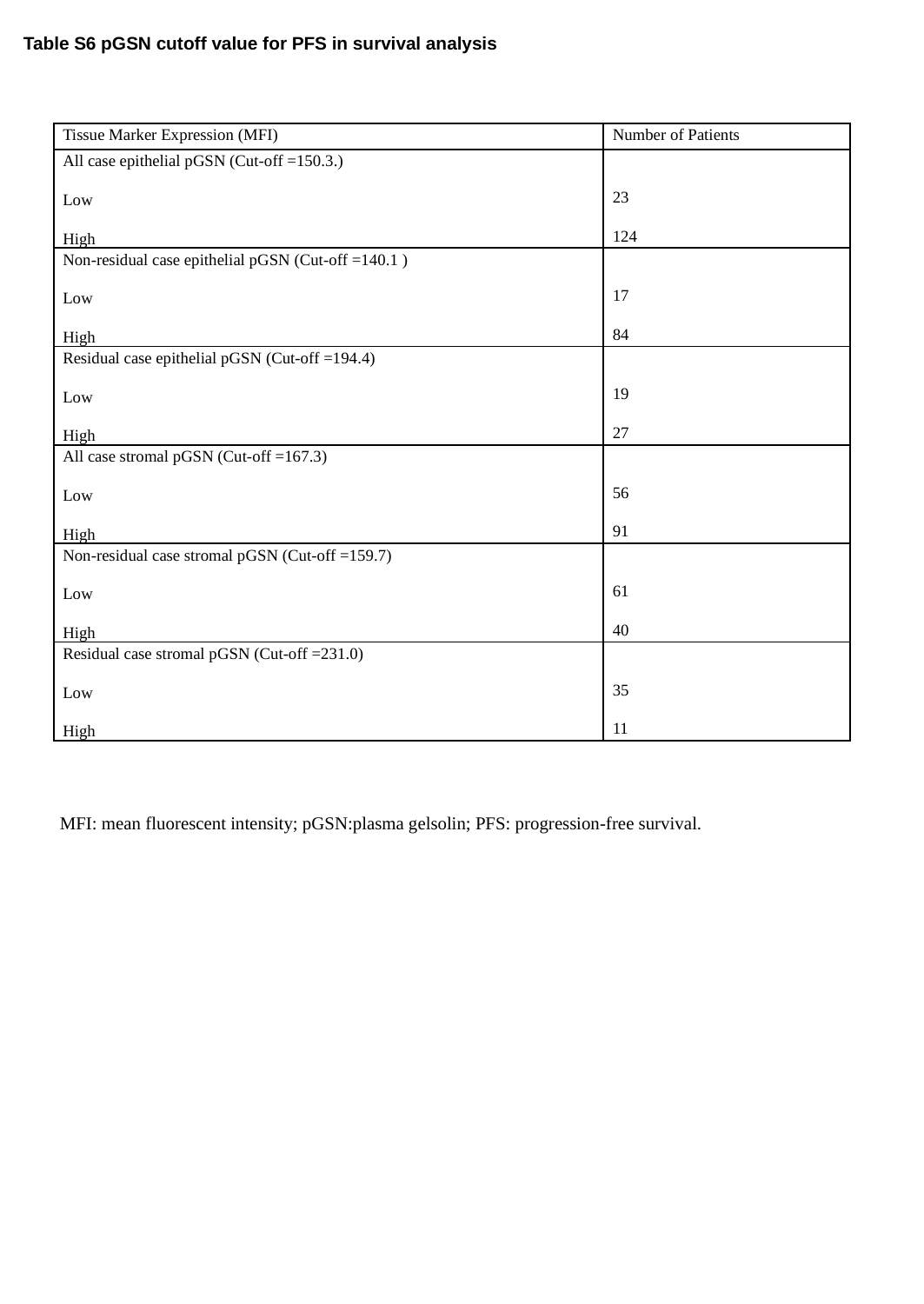

Table S6 pGSN cutoff value for PFS in survival analysis
| Tissue Marker Expression (MFI) | Number of Patients |
| --- | --- |
| All case epithelial pGSN (Cut-off =150.3.) Low High | 23 124 |
| Non-residual case epithelial pGSN (Cut-off =140.1 ) Low High | 17 84 |
| Residual case epithelial pGSN (Cut-off =194.4) Low High | 19 27 |
| All case stromal pGSN (Cut-off =167.3) Low High | 56 91 |
| Non-residual case stromal pGSN (Cut-off =159.7) Low High | 61 40 |
| Residual case stromal pGSN (Cut-off =231.0) Low High | 35 11 |
MFI: mean fluorescent intensity; pGSN:plasma gelsolin; PFS: progression-free survival.

## Slide 6
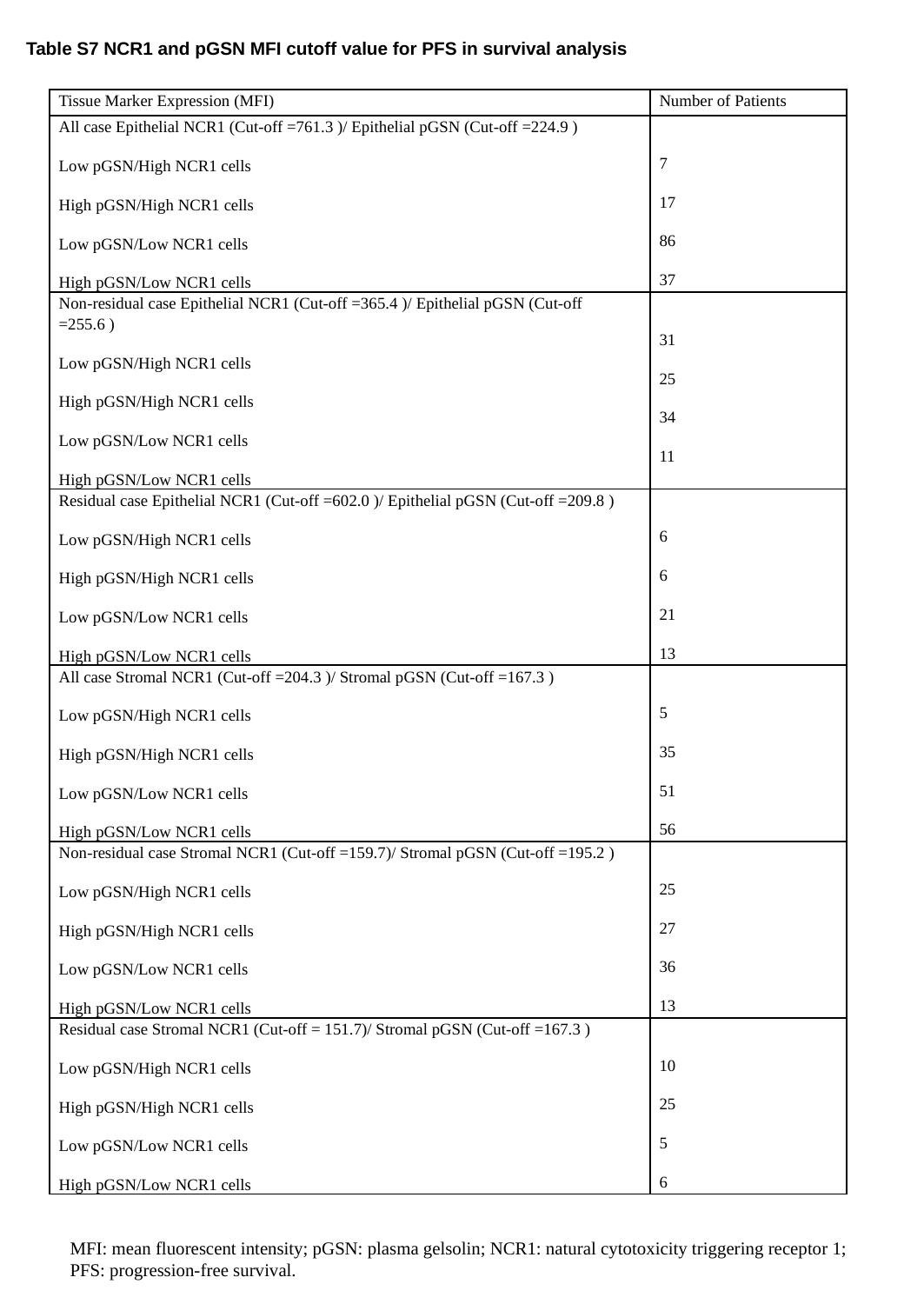

Table S7 NCR1 and pGSN MFI cutoff value for PFS in survival analysis
| Tissue Marker Expression (MFI) | Number of Patients |
| --- | --- |
| All case Epithelial NCR1 (Cut-off =761.3 )/ Epithelial pGSN (Cut-off =224.9 ) Low pGSN/High NCR1 cells High pGSN/High NCR1 cells Low pGSN/Low NCR1 cells High pGSN/Low NCR1 cells | 7 17 86 37 |
| Non-residual case Epithelial NCR1 (Cut-off =365.4 )/ Epithelial pGSN (Cut-off =255.6 ) Low pGSN/High NCR1 cells High pGSN/High NCR1 cells Low pGSN/Low NCR1 cells High pGSN/Low NCR1 cells | 31 25 34 11 |
| Residual case Epithelial NCR1 (Cut-off =602.0 )/ Epithelial pGSN (Cut-off =209.8 ) Low pGSN/High NCR1 cells High pGSN/High NCR1 cells Low pGSN/Low NCR1 cells High pGSN/Low NCR1 cells | 6 6 21 13 |
| All case Stromal NCR1 (Cut-off =204.3 )/ Stromal pGSN (Cut-off =167.3 ) Low pGSN/High NCR1 cells High pGSN/High NCR1 cells Low pGSN/Low NCR1 cells High pGSN/Low NCR1 cells | 5 35 51 56 |
| Non-residual case Stromal NCR1 (Cut-off =159.7)/ Stromal pGSN (Cut-off =195.2 ) Low pGSN/High NCR1 cells High pGSN/High NCR1 cells Low pGSN/Low NCR1 cells High pGSN/Low NCR1 cells | 25 27 36 13 |
| Residual case Stromal NCR1 (Cut-off = 151.7)/ Stromal pGSN (Cut-off =167.3 ) Low pGSN/High NCR1 cells High pGSN/High NCR1 cells Low pGSN/Low NCR1 cells High pGSN/Low NCR1 cells | 10 25 5 6 |
MFI: mean fluorescent intensity; pGSN: plasma gelsolin; NCR1: natural cytotoxicity triggering receptor 1; PFS: progression-free survival.

## Slide 7
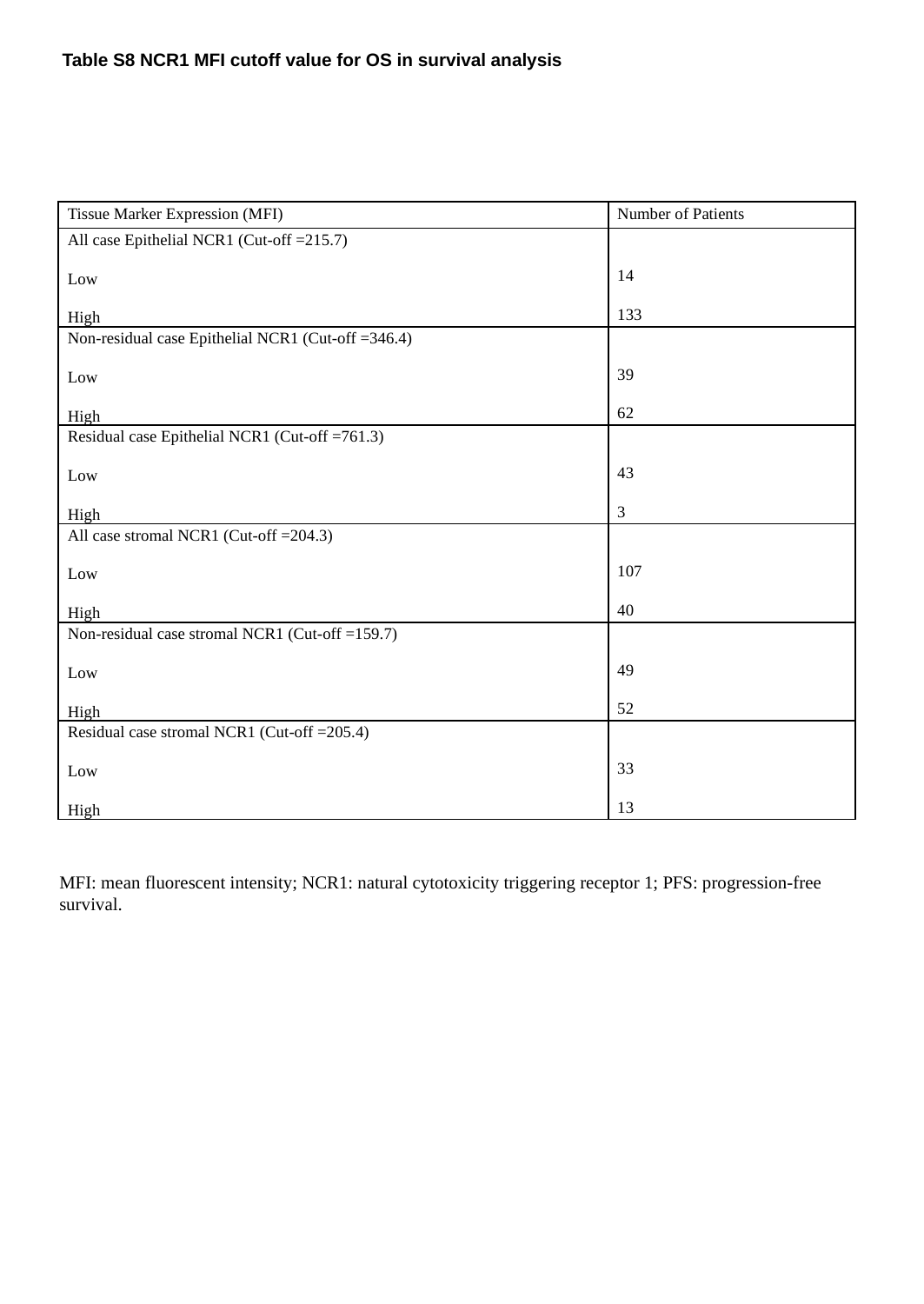

Table S8 NCR1 MFI cutoff value for OS in survival analysis
| Tissue Marker Expression (MFI) | Number of Patients |
| --- | --- |
| All case Epithelial NCR1 (Cut-off =215.7) Low High | 14 133 |
| Non-residual case Epithelial NCR1 (Cut-off =346.4) Low High | 39 62 |
| Residual case Epithelial NCR1 (Cut-off =761.3) Low High | 43 3 |
| All case stromal NCR1 (Cut-off =204.3) Low High | 107 40 |
| Non-residual case stromal NCR1 (Cut-off =159.7) Low High | 49 52 |
| Residual case stromal NCR1 (Cut-off =205.4) Low High | 33 13 |
MFI: mean fluorescent intensity; NCR1: natural cytotoxicity triggering receptor 1; PFS: progression-free survival.

## Slide 8
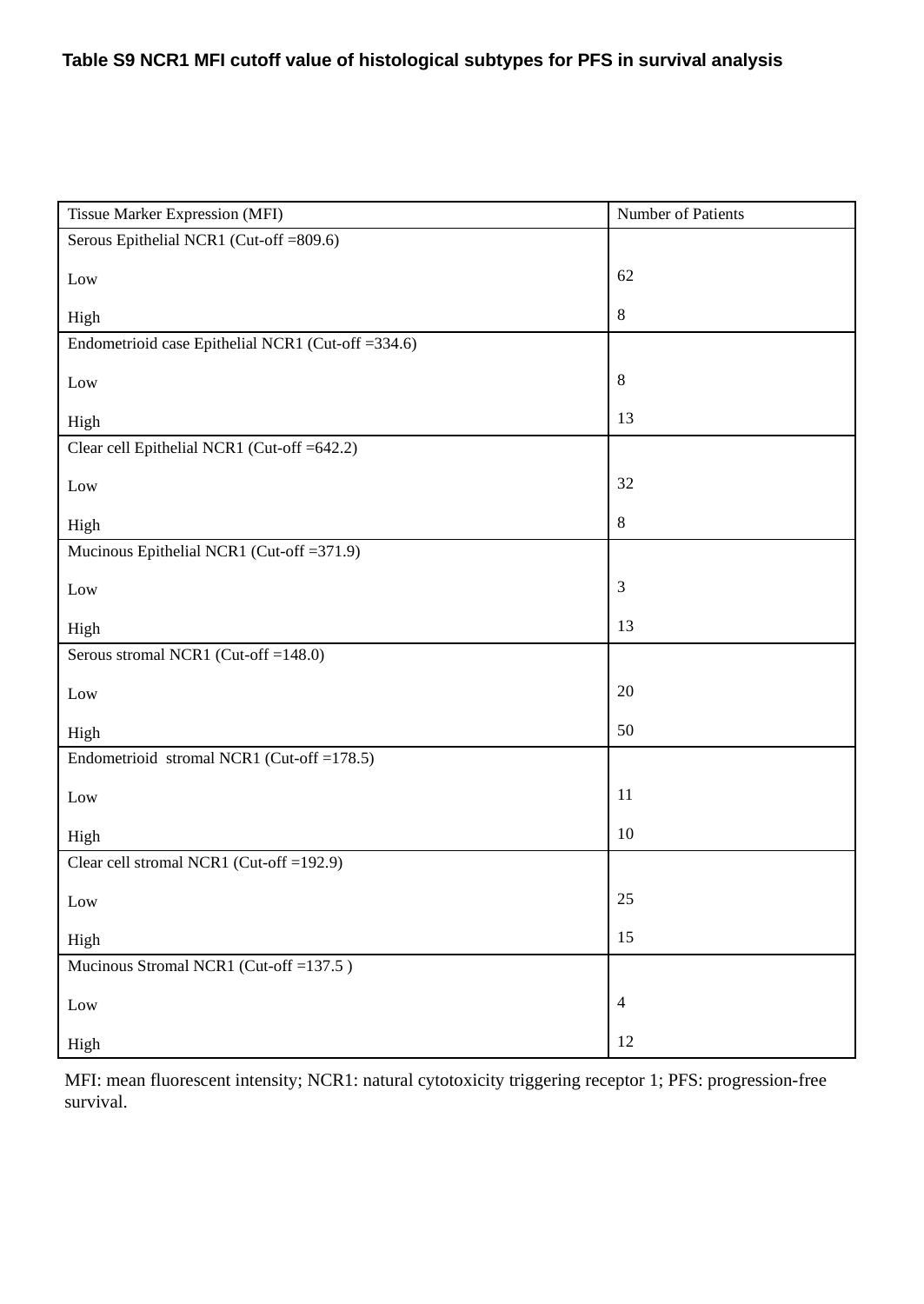

Table S9 NCR1 MFI cutoff value of histological subtypes for PFS in survival analysis
| Tissue Marker Expression (MFI) | Number of Patients |
| --- | --- |
| Serous Epithelial NCR1 (Cut-off =809.6) Low High | 62 8 |
| Endometrioid case Epithelial NCR1 (Cut-off =334.6) Low High | 8 13 |
| Clear cell Epithelial NCR1 (Cut-off =642.2) Low High | 32 8 |
| Mucinous Epithelial NCR1 (Cut-off =371.9) Low High | 3 13 |
| Serous stromal NCR1 (Cut-off =148.0) Low High | 20 50 |
| Endometrioid stromal NCR1 (Cut-off =178.5) Low High | 11 10 |
| Clear cell stromal NCR1 (Cut-off =192.9) Low High | 25 15 |
| Mucinous Stromal NCR1 (Cut-off =137.5 ) Low High | 4 12 |
MFI: mean fluorescent intensity; NCR1: natural cytotoxicity triggering receptor 1; PFS: progression-free survival.

## Slide 9
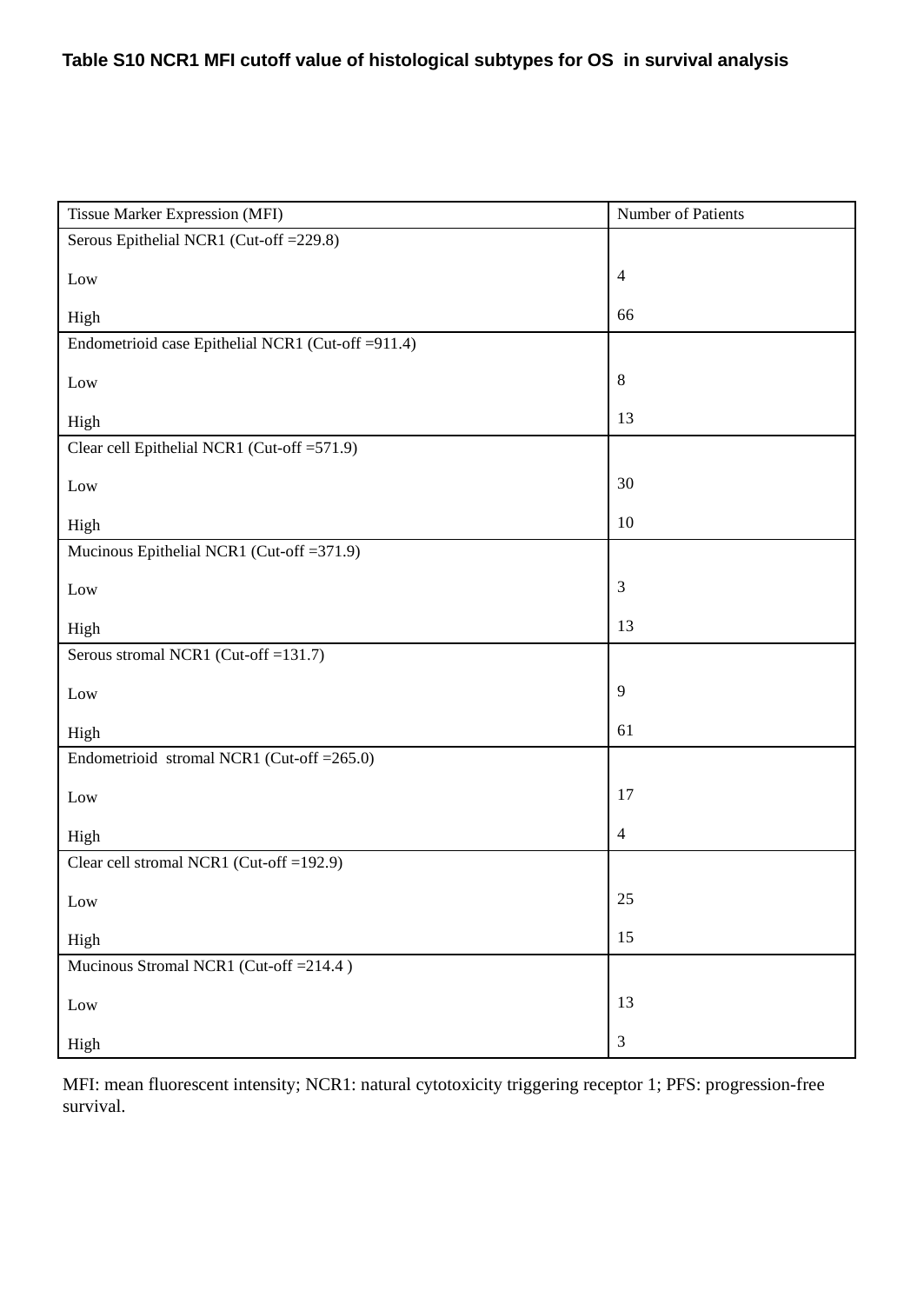

Table S10 NCR1 MFI cutoff value of histological subtypes for OS in survival analysis
| Tissue Marker Expression (MFI) | Number of Patients |
| --- | --- |
| Serous Epithelial NCR1 (Cut-off =229.8) Low High | 4 66 |
| Endometrioid case Epithelial NCR1 (Cut-off =911.4) Low High | 8 13 |
| Clear cell Epithelial NCR1 (Cut-off =571.9) Low High | 30 10 |
| Mucinous Epithelial NCR1 (Cut-off =371.9) Low High | 3 13 |
| Serous stromal NCR1 (Cut-off =131.7) Low High | 9 61 |
| Endometrioid stromal NCR1 (Cut-off =265.0) Low High | 17 4 |
| Clear cell stromal NCR1 (Cut-off =192.9) Low High | 25 15 |
| Mucinous Stromal NCR1 (Cut-off =214.4 ) Low High | 13 3 |
MFI: mean fluorescent intensity; NCR1: natural cytotoxicity triggering receptor 1; PFS: progression-free survival.

## Slide 10
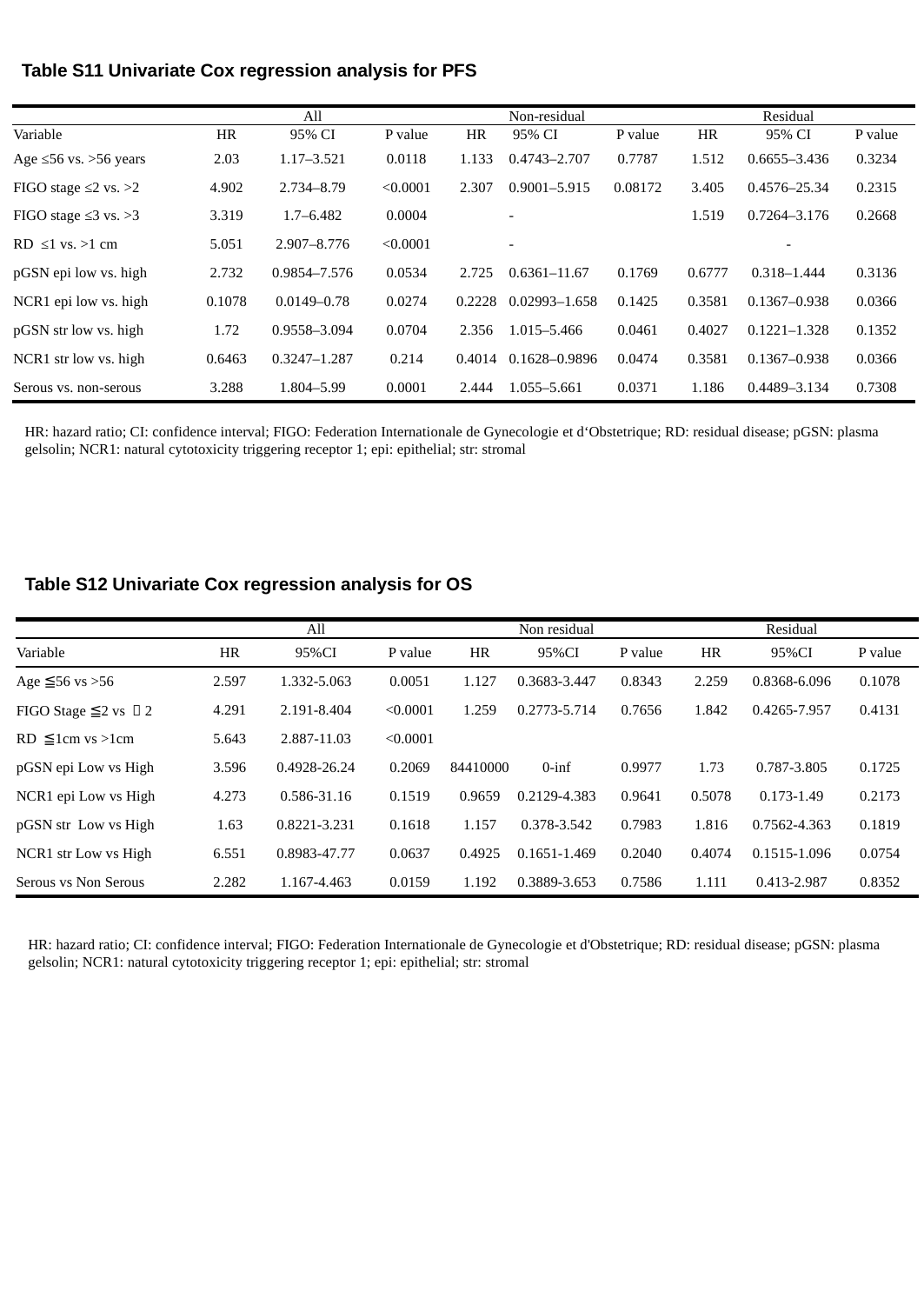

Table S11 Univariate Cox regression analysis for PFS
| | | All | | | Non-residual | | | Residual | |
| --- | --- | --- | --- | --- | --- | --- | --- | --- | --- |
| Variable | HR | 95% CI | P value | HR | 95% CI | P value | HR | 95% CI | P value |
| Age ≤56 vs. >56 years | 2.03 | 1.17–3.521 | 0.0118 | 1.133 | 0.4743–2.707 | 0.7787 | 1.512 | 0.6655–3.436 | 0.3234 |
| FIGO stage ≤2 vs. >2 | 4.902 | 2.734–8.79 | <0.0001 | 2.307 | 0.9001–5.915 | 0.08172 | 3.405 | 0.4576–25.34 | 0.2315 |
| FIGO stage ≤3 vs. >3 | 3.319 | 1.7–6.482 | 0.0004 | | - | | 1.519 | 0.7264–3.176 | 0.2668 |
| RD ≤1 vs. >1 cm | 5.051 | 2.907–8.776 | <0.0001 | | - | | | - | |
| pGSN epi low vs. high | 2.732 | 0.9854–7.576 | 0.0534 | 2.725 | 0.6361–11.67 | 0.1769 | 0.6777 | 0.318–1.444 | 0.3136 |
| NCR1 epi low vs. high | 0.1078 | 0.0149–0.78 | 0.0274 | 0.2228 | 0.02993–1.658 | 0.1425 | 0.3581 | 0.1367–0.938 | 0.0366 |
| pGSN str low vs. high | 1.72 | 0.9558–3.094 | 0.0704 | 2.356 | 1.015–5.466 | 0.0461 | 0.4027 | 0.1221–1.328 | 0.1352 |
| NCR1 str low vs. high | 0.6463 | 0.3247–1.287 | 0.214 | 0.4014 | 0.1628–0.9896 | 0.0474 | 0.3581 | 0.1367–0.938 | 0.0366 |
| Serous vs. non-serous | 3.288 | 1.804–5.99 | 0.0001 | 2.444 | 1.055–5.661 | 0.0371 | 1.186 | 0.4489–3.134 | 0.7308 |
HR: hazard ratio; CI: confidence interval; FIGO: Federation Internationale de Gynecologie et d‘Obstetrique; RD: residual disease; pGSN: plasma gelsolin; NCR1: natural cytotoxicity triggering receptor 1; epi: epithelial; str: stromal
Table S12 Univariate Cox regression analysis for OS
| | | All | | | Non residual | | | Residual | |
| --- | --- | --- | --- | --- | --- | --- | --- | --- | --- |
| Variable | HR | 95%CI | P value | HR | 95%CI | P value | HR | 95%CI | P value |
| Age ≦56 vs >56 | 2.597 | 1.332-5.063 | 0.0051 | 1.127 | 0.3683-3.447 | 0.8343 | 2.259 | 0.8368-6.096 | 0.1078 |
| FIGO Stage ≦2 vs ＞2 | 4.291 | 2.191-8.404 | <0.0001 | 1.259 | 0.2773-5.714 | 0.7656 | 1.842 | 0.4265-7.957 | 0.4131 |
| RD ≦1cm vs >1cm | 5.643 | 2.887-11.03 | <0.0001 | | | | | | |
| pGSN epi Low vs High | 3.596 | 0.4928-26.24 | 0.2069 | 84410000 | 0-inf | 0.9977 | 1.73 | 0.787-3.805 | 0.1725 |
| NCR1 epi Low vs High | 4.273 | 0.586-31.16 | 0.1519 | 0.9659 | 0.2129-4.383 | 0.9641 | 0.5078 | 0.173-1.49 | 0.2173 |
| pGSN str Low vs High | 1.63 | 0.8221-3.231 | 0.1618 | 1.157 | 0.378-3.542 | 0.7983 | 1.816 | 0.7562-4.363 | 0.1819 |
| NCR1 str Low vs High | 6.551 | 0.8983-47.77 | 0.0637 | 0.4925 | 0.1651-1.469 | 0.2040 | 0.4074 | 0.1515-1.096 | 0.0754 |
| Serous vs Non Serous | 2.282 | 1.167-4.463 | 0.0159 | 1.192 | 0.3889-3.653 | 0.7586 | 1.111 | 0.413-2.987 | 0.8352 |
HR: hazard ratio; CI: confidence interval; FIGO: Federation Internationale de Gynecologie et d'Obstetrique; RD: residual disease; pGSN: plasma gelsolin; NCR1: natural cytotoxicity triggering receptor 1; epi: epithelial; str: stromal
